# Diagnosis and molecular characterization of rabies virus from a buffalo in China: a case report

**DOI:** 10.1186/1743-422X-8-101

**Published:** 2011-03-06

**Authors:** Ke-shan Zhang, Jian-hong Guo, Zhuo-fei Xu, Min Xiang, Bin Wu, Huan-chun Chen

**Affiliations:** 1 State Key Laboratory of Veterinary Etiological Biology, National Foot-and-Mouth Disease Reference Laboratory, Key Laboratory of Animal Virology of Ministry of Agriculture, Lanzhou Veterinary Research Institute of Chinese Academy of Agriculture Science, Xujiaping No.1, Yanchangpu, Lanzhou, Gansu, 730046, PR China; 2 State Key Laboratory of Agricultural Microbiology, College of Veterinary Medicine, Huazhong Agricultural University, Wuhan 430070, Hubei, PR China

## Abstract

**Background:**

Rabies virus (RABV) can infect many different species of warm-blooded animals. Glycoprotein G plays a key role in viral pathogenicity and neurotropism, and includes antigenic domains that are responsible for membrane fusion and host cell receptor recognition.

**Case presentation:**

A case of buffalo rabies in China was diagnosed by direct fluorescent antibody test, G gene reverse-transcriptase polymerase chain reaction, and RABV mouse inoculation test. Molecular characterization of the RABV was performed using DNA sequencing, phylogenetic analysis and amino acid sequence comparison based on the G gene from different species of animals.

**Conclusion:**

The results confirmed that the buffalo with suspected rabies was infected by RABV, which was genetically closely related to HNC (FJ602451) that was isolated from cattle in China in 2007. Comparison of the G gene among different species of animal showed that there were almost no amino acid changes among RABVs isolated from the same species of animals that distributed in a near region. However, there were many changes among RABVs that were isolated from different species of animal, or the same species from different geographic regions. This is believed to be the first case report of buffalo rabies in China, and the results may provide further information to understand the mechanism by which RABV breaks through the species barrier.

## Background

Rabies virus (RABV) is one of the seven species in the genus Lyssavirus in the Rhabdoviridae family [1]. All warm-blooded animals, including raccoons, skunks, bats and foxes, are susceptible to RABV, and domestic dogs act as the main reservoir and transmitter [2]. The annual number of human deaths caused by rabies is estimated to be 55,000 worldwide [3], with about 32,000 in Asia [4]. The total number of human deaths was 108,412 between 1950 and 2004 in China [5]. The average number was 1,524 from 1996 to 2008, and 50% of cases were reported in Guangxi, Hunan and Guizhou provinces [6]. Therefore, the disease continues to be a serious public and animal health problem in China.

RABV has a non-segmented, negative-sense, single-stranded RNA genome about 12 kb in length [7], with five genes: L (RNA polymerase), G (glycoprotein), M (matrix protein), P (phosphoprotein), and N (nucleoprotein) [8]. The order of relative conservation of these five genes from high to low could be either N>L>M>P>G, or N>L>M>G>P[9]. Glycoprotein G of RABV plays an important role in pathogenicity [10,11] and viral neurotropism [12] because it contains membrane fusion sites [13] and host cell receptor domains [14,15]. In recent years, molecular epidemiology [16-18] and diagnosis [19,20] of rabies have been based on the G gene.

The aims of the present study were to diagnose a case of buffalo rabies that occurred in Wuhan City, Hubei Province, using three different methods, and to compare the sequences with different RABVs that were isolated from different species, based on the G gene. This is believed to be the first report of the phylogenetic analysis of buffalo RABV in China compared with other isolates from different animals.

## Case presentation

Specimens were collected from the gyrus hippocampi of the buffalo with suspected rabies in Wuhan City (114.3°E, 30.8°N), Hubei Province, China. Direct fluorescent antibody test (dFAT) of the specimen was performed as previously described [21-23]. Normal buffalo brain samples were used as a negative control. RABV isolation by mouse inoculation test was performed as described previously [24,25].

The total RNA from buffalo brain was extracted with Trizol reagent (Invitrogen) according to the manufacturer's instructions. Primer design and reverse-transcriptase polymerase chain reaction (RT-PCR) of the G gene were performed as described previously [17]. RT-PCR products were visualized under UV light after electrophoresis on 1% agarose gels containing ethidium bromide. The amplified products were purified with a QIAquick PCR gel extraction kit (QIAGEN) according to the manufacturer's protocol. The sequencing was carried out in an Applied Biosystems 3730 DNA automated sequencer. After the raw sequences were edited by ClustalX Version 1.82 [26], 1575 nt sequences of the G gene were obtained and submitted to GenBank.

The phylogenetic tree based on the deduced amino acid sequences was constructed by using the neighbor-joining method with 1,000 bootstrap replicates using MEGA version 4.0 software [27], based on the complete sequence of the RABV G gene from 10 different species of animal (Table 1). Bootstrap values >70% were considered significant [28]. Genetic distance analysis for the G gene was conducted with PHYLIP version 3.63 software [29]. Glycoprotein nucleotide sequences from different animals were identified, translated into amino acid sequences, edited, and pair aligned using BioEdit software [27]. Multiple alignments were performed by ClustalX software [26].

**Table 1:** Detailed information of the G gene used in the present study

**No**.	Virus strains	Country and year of isolation	Accession Number	Host species
1	**NNV-RAB-H**	**India 2007**	EF437215	homo sapiens
2	**ZAMRAV51/00**	**Zambia 2000**	AB285215	dog
3	**HN06**	**China 2005**	DQ849062	dog
4	**FY3**	**China 2004**	DQ849046	dog
5	**THA1-HM**	**Thailand 1983**	AF325488	human
6	**MAL1-HM**	**Malaysia 1985**	AF325487	human
7	**CHI1-BK**	**China 1986**	AF325471	deer
8	**COSRV**	**USA 1996**	U52947	coyote
9	**SHBRV**	**USA 1996**	U52946	bat
10	**90RABN5850**	**Canada 2001**	U11754	vulpes
11	**PA R89**	**Canada 2001**	U27217	Procyon
12	**92RBG1741**	**Canada 2001**	AF344305	skunk
13	**Hubei070308**	**China 2007**	EF643518	buffalo
14	**PG**	**China 1931**	AY009097	dog(Vaccine Strain)
15	**QC**	**China 2006**	DQ849063	human
16	**HNC**	**China 2007**	FJ602451	cattle
17	**LuoH**	**China 2007**	FJ602453	Homo sapiens
18	**92RBGL0867**	**Canada 1992**	AF344307	Striped skunk
**19**	NY516	USA 1995	U27214	raccoon

## Conclusion

**Figure 1:**
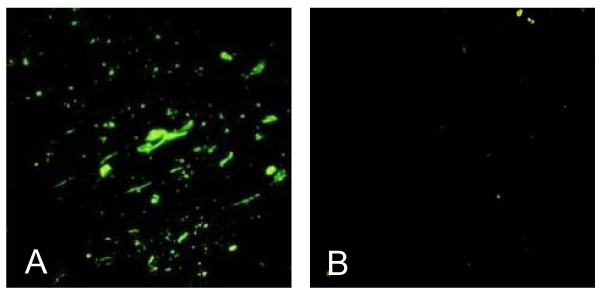
**Results of dFAT of specimens from brain of buffalo with suspected rabies**. (A) suspected buffalo brain sample; (B) normal buffalo brain sample.

dFAT indicated the presence of RABV antigen in the brain specimens from the buffalo with suspected rabies (Figure 1A), whereas normal buffalo brain did not (Figure 1B). The expected size of the G gene fragment was obtained from the suspected buffalo brain by RT-PCR (data not shown). RABV from buffalo was isolated successfully in suckling mice (data not shown), and the RABV was named Hubei070308 strain. The positive result was supported by G gene sequencing, and the sequences were submitted to GenBank under the accession number EF643518.

**Figure 2:**
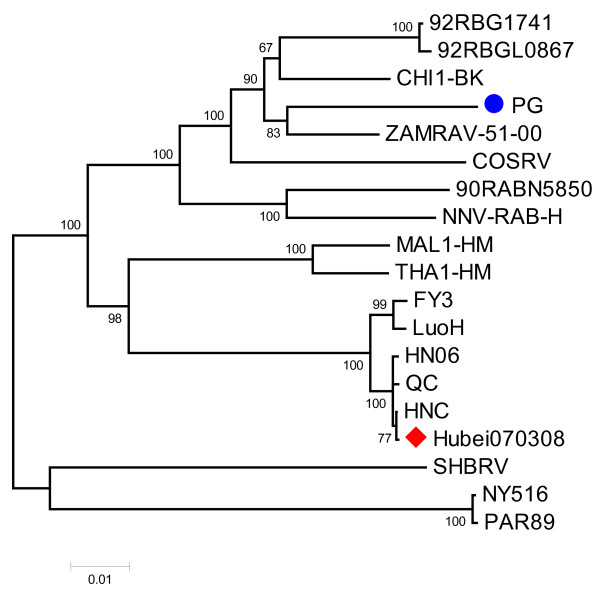
**Phylogenetic analysis based on deduced amino acid sequence of complete G gene**. The phylogenetic tree was constructed by the neighbor-joining algorithm using MEGA version 4.0, and bootstrap analysis was performed with 1,000 trials. All sequences were collected from GenBank. The red spot indicates Hubei070308 that was isolated in the present study. The blue spot indicates the 1931 RABV vaccine strain.

The G gene sequence, together with reference sequences from seven countries and 10 species of animals were aligned (Table 1). We showed that the G gene of RABV had relative territorial specificity but not species specificity (Figure 2). The genetic relationship of the RABV in this study was differed greatly from PG(AY009097) that was used as a vaccine strain in China in 1931. Compared with 18 other RABV strains, Hubei070308 shared 85.0-99.8% sequence identity at the amino acid level (data not shown). We demonstrated that Hubei070308 strain was close to the cattle strain FJ602451 and the human strain DQ849063, which belong to Chinese group I [17], but it was far from coyote strain U52946, which was isolated in 1996 in the United States (Figure 2).

Additional File 1, Figure S1 shows that the amino acid changes were mainly focused on the transmembrane areas (aa 440-461), inner-membrane zone (aa 462-505) and signal peptide range of mature RABV glycoprotein. Glycoprotein sequences of Hubei070308 were identical to HNC(FJ602451) that was isolated from cattle in 2007. However, there were many amino acid substitutions when it was compared with RABVs from other animals such as skunk, dog, human, bat and deer. The linear epitope (aa 14-19) at antigenic site II and the minor site between aa 342 and 343 were highly conserved, which was consistent with the findings of Meng *et al*. [17]. Among all the RABVs, SHBRV strain that was isolated from bats was the most variable at the amino acid level. Many different animals can be infected by RABV [2], and cases of transmission from bats to humans [30], dogs to humans [17] and even dogs to pigs [31] have been reported. For a virus shed by one host to infect another, it must break through entry barriers (e.g., epithelium, mucus, and alveolar macrophages) and find its way to tissues in which it can replicate [32].

It has been reported that several amino acids in the RABV glycoprotein are responsible for pathogenicity [33,34]. Therefore, RABV glycoprotein is the best target protein to study virus-host interaction, or it may be the main protein that is responsible for breakthrough of the species barrier. In the present study, many amino acid substitutions in G protein were found among RABVs that were isolated from different animal species, or from the same species distributed in different geographic regions. These substituted amino acids may explain why RABV can break through the host barrier to infect one species of animal from another. This hypothesis needs to be confirmed by further experiments.

## List of abbreviations

dFAT: direct fluorescent antibody test (dFAT); G: Glycoprotein; RABV: rabies virus; RT-RCR: reverse-transcriptase polymerase chain reaction.

## Competing interests

The authors declare that they have no competing interests.

## Authors' contributions

BW was the leader of the project. KZ carried out most of the studies and drafted the manuscript. ZX and MX amplified the complete G gene. HC and JG provided consultation and preparation of the final report. All authors read and approved the final manuscript.

## Supplementary Material

Additional file 1 **Figure S1. Comparative analysis of G gene amino acid with other RABVs isolated from different animals**. Dots represent identity among all sequences. Arrows mark the range of the signal peptide, antigenic site, linear epitope and endo-domain. Trans-membrane (TM) domain was framed. SP: signal peptides; ENDO: endo-domain; AS2: antigenic site II; AS3: antigenic site III; LE: linear epitope. Click here for file

## References

[B1] Mayo MA Haenni AL Report from the 36th and the 37th meetings of the Executive Committee of the International Committee on Taxonomy of Viruses Arch Virol 2006 151 1031 1037 10.1007/s00705-006-0728-9 16514500 PMC7086863

[B2] Cisterna D Bonaventura R Caillou S Pozo O Andreau ML Fontana LD Echegoyen C de Mattos C de Mattos C Russo S Antigenic and molecular characterization of rabies virus in Argentina Virus Res 2005 109 139 147 10.1016/j.virusres.2004.10.013 15763144

[B3] Knobel DL Cleaveland S Coleman PG Fevre EM Meltzer MI Miranda ME Shaw A Zinsstag J Meslin FX Re-evaluating the burden of rabies in Africa and Asia Bull World Health Organ 2005 83 360 368 15976877 PMC2626230

[B4] Sugiyama M Ito N Control of rabies: epidemiology of rabies in Asia and development of new-generation vaccines for rabies Comp Immunol Microbiol Infect Dis 2007 30 273 286 10.1016/j.cimid.2007.05.007 17619057

[B5] Zhang YZ Xiong CL Xiao DL Jiang RJ Wang ZX Zhang LZ Fu ZF Human rabies in China Emerg Infect Dis 2005 11 1983 1984 16485502 10.3201/eid1112.040775 PMC3367615

[B6] Song M Tang Q Wang DM Mo ZJ Guo SH Li H Tao XY Rupprecht CE Feng ZJ Liang GD Epidemiological investigations of human rabies in China BMC Infect Dis 2009 9 210 10.1186/1471-2334-9-210 20025742 PMC2803182

[B7] Bourhy H Kissi B Tordo N Molecular diversity of the Lyssavirus genus Virology 1993 194 70 81 10.1006/viro.1993.1236 8386891

[B8] Shimizu K Ito N Mita T Yamada K Hosokawa-Muto J Sugiyama M Minamoto N Involvement of nucleoprotein, phosphoprotein, and matrix protein genes of rabies virus in virulence for adult mice Virus Res 2007 123 154 160 10.1016/j.virusres.2006.08.011 17010466

[B9] Wu X Franka R Velasco-Villa A Rupprecht CE Are all lyssavirus genes equal for phylogenetic analyses? Virus Res 2007 129 91 103 10.1016/j.virusres.2007.06.022 17681631

[B10] Morimoto K Hooper DC Spitsin S Koprowski H Dietzschold B Pathogenicity of different rabies virus variants inversely correlates with apoptosis and rabies virus glycoprotein expression in infected primary neuron cultures J Virol 1999 73 510 518 9847357 10.1128/jvi.73.1.510-518.1999 PMC103858

[B11] Faber M Faber ML Papaneri A Bette M Weihe E Dietzschold B Schnell MJ A single amino acid change in rabies virus glycoprotein increases virus spread and enhances virus pathogenicity J Virol 2005 79 14141 14148 10.1128/JVI.79.22.14141-14148.2005 16254349 PMC1280225

[B12] Badrane H Bahloul C Perrin P Tordo N Evidence of two Lyssavirus phylogroups with distinct pathogenicity and immunogenicity J Virol 2001 75 3268 3276 10.1128/JVI.75.7.3268-3276.2001 11238853 PMC114120

[B13] Durrer P Gaudin Y Ruigrok RW Graf R Brunner J Photolabeling identifies a putative fusion domain in the envelope glycoprotein of rabies and vesicular stomatitis viruses J Biol Chem 1995 270 17575 17581 10.1074/jbc.270.29.17575 7615563

[B14] Thoulouze MI Lafage M Schachner M Hartmann U Cremer H Lafon M The neural cell adhesion molecule is a receptor for rabies virus J Virol 1998 72 7181 7190 9696812 10.1128/jvi.72.9.7181-7190.1998 PMC109940

[B15] Tuffereau C Benejean J Blondel D Kieffer B Flamand A Low-affinity nerve-growth factor receptor (P75NTR) can serve as a receptor for rabies virus Embo J 1998 17 7250 7259 10.1093/emboj/17.24.7250 9857182 PMC1171071

[B16] Zhang S Zhao J Liu Y Fooks AR Zhang F Hu R Characterization of a rabies virus isolate from a ferret badger (Melogale moschata) with unique molecular differences in glycoprotein antigenic site III Virus Res 2010 149 143 151 10.1016/j.virusres.2010.01.010 20109507

[B17] Meng SL Yan JX Xu GL Nadin-Davis SA Ming PG Liu SY Wu J Ming HT Zhu FC Zhou DJ A molecular epidemiological study targeting the glycoprotein gene of rabies virus isolates from China Virus Res 2007 124 125 138 10.1016/j.virusres.2006.10.011 17129631

[B18] Carnieli P Jr Castilho JG Fahl Wde O Veras NM Carrieri ML Kotait I Molecular characterization of Rabies Virus isolates from dogs and crab-eating foxes in Northeastern Brazil Virus Res 2009 141 81 89 10.1016/j.virusres.2008.12.015 19185599

[B19] Feyssaguet M Dacheux L Audry L Compoint A Morize JL Blanchard I Bourhy H Multicenter comparative study of a new ELISA, PLATELIA RABIES II, for the detection and titration of anti-rabies glycoprotein antibodies and comparison with the rapid fluorescent focus inhibition test (RFFIT) on human samples from vaccinated and non-vaccinated people Vaccine 2007 25 2244 2251 10.1016/j.vaccine.2006.12.012 17224214

[B20] Flamand A Wiktor TJ Koprowski H Use of hybridoma monoclonal antibodies in the detection of antigenic differences between rabies and rabies-related virus proteins. II. The glycoprotein J Gen Virol 1980 48 105 109 10.1099/0022-1317-48-1-105 6155432

[B21] Rudd RJ Smith JS Yager PA Orciari LA Trimarchi CV A need for standardized rabies-virus diagnostic procedures: effect of cover-glass mountant on the reliability of antigen detection by the fluorescent antibody test Virus Res 2005 111 83 88 10.1016/j.virusres.2005.03.014 15896406

[B22] Tepsumethanon V Lumlertdacha B Mitmoonpitak C Fagen R Wilde H Fluorescent antibody test for rabies: prospective study of 8,987 brains Clin Infect Dis 1997 25 1459 1461 10.1086/516151 9431394

[B23] Bingham J van der Merwe M Distribution of rabies antigen in infected brain material: determining the reliability of different regions of the brain for the rabies fluorescent antibody test J Virol Methods 2002 101 85 94 10.1016/S0166-0934(01)00423-2 11849687

[B24] Chhabra M Bhardwaj M Ichhpujani RL Lal S Comparative evaluation of commonly used laboratory tests for post-mortem diagnosis of rabies Indian J Pathol Microbiol 2005 48 190 193 16758661

[B25] Chhabra M Mittal V Jaiswal R Malik S Gupta M Lal S Development and evaluation of an in vitro isolation of street rabies virus in mouse neuroblastoma cells as compared to conventional tests used for diagnosis of rabies Indian J Med Microbiol 2007 25 263 266 10.4103/0255-0857.34772 17901648

[B26] Thompson JD Gibson TJ Plewniak F Jeanmougin F Higgins DG The CLUSTAL_X windows interface: flexible strategies for multiple sequence alignment aided by quality analysis tools Nucleic Acids Res 1997 25 4876 4882 10.1093/nar/25.24.4876 9396791 PMC147148

[B27] Tamura K Dudley J Nei M Kumar S MEGA4: Molecular Evolutionary Genetics Analysis (MEGA) software version 4.0 Mol Biol Evol 2007 24 1596 1599 10.1093/molbev/msm092 17488738

[B28] Beasley WH Deshea L Toothaker LE Mendoza JL Bard DE Rodgers JL Bootstrapping to test for nonzero population correlation coefficients using univariate sampling Psychol Methods 2007 12 414 433 10.1037/1082-989X.12.4.414 18179352

[B29] Retief JD Phylogenetic analysis using PHYLIP Methods Mol Biol 2000 132 243 258 10547839 10.1385/1-59259-192-2:243

[B30] da Rosa ES Kotait I Barbosa TF Carrieri ML Brandao PE Pinheiro AS Begot AL Wada MY de Oliveira RC Grisard EC Bat-transmitted human rabies outbreaks, Brazilian Amazon Emerg Infect Dis 2006 12 1197 1202 16965697 10.3201/1208.050929 PMC3291204

[B31] Jiang Y Yu X Wang L Lu Z Liu H Xuan H Hu Z Tu C An outbreak of pig rabies in Hunan province, China Epidemiol Infect 2008 136 504 508 10.1017/S0950268807008874 17559696 PMC2870836

[B32] Kuiken T Holmes EC McCauley J Rimmelzwaan GF Williams CS Grenfell BT Host species barriers to influenza virus infections Science 2006 312 394 397 10.1126/science.1122818 16627737

[B33] Takayama-Ito M Ito N Yamada K Sugiyama M Minamoto N Multiple amino acids in the glycoprotein of rabies virus are responsible for pathogenicity in adult mice Virus Res 2006 115 169 175 10.1016/j.virusres.2005.08.004 16188341

[B34] Takayama-Ito M Ito N Yamada K Minamoto N Sugiyama M Region at amino acids 164 to 303 of the rabies virus glycoprotein plays an important role in pathogenicity for adult mice J Neurovirol 2004 10 131 135 10.1080/13550280490279799 15204932

