# Supplementary material for: Diagnosis and molecular characterization of rabies virus from a buffalo in China: a case report
Source: Virol J. 2011 Mar 6;8:101. doi: 10.1186/1743-422X-8-101 (PMC3061937; doi:10.1186/1743-422X-8-101)
Supplement: Additional file 1 — Figure S1. Comparative analysis of G gene amino acid with other RABVs isolated from different animals. Dots represent identity among all sequences. Arrows mark the range of the signal peptide, antigenic site, linear epitope and endo-domain. Trans-membrane (TM) domain was framed. SP: signal peptides; ENDO: endo-domain; AS2: antigenic site II; AS3: antigenic site III; LE: linear epitope. [file 1743-422X-8-101-S1.DOC]

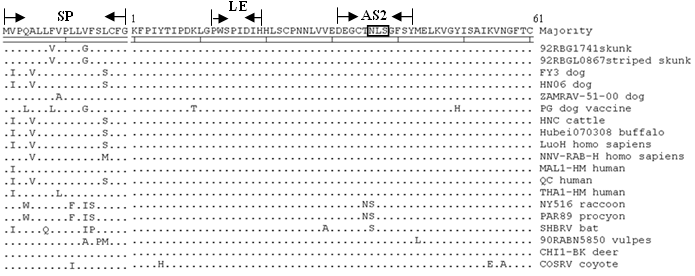


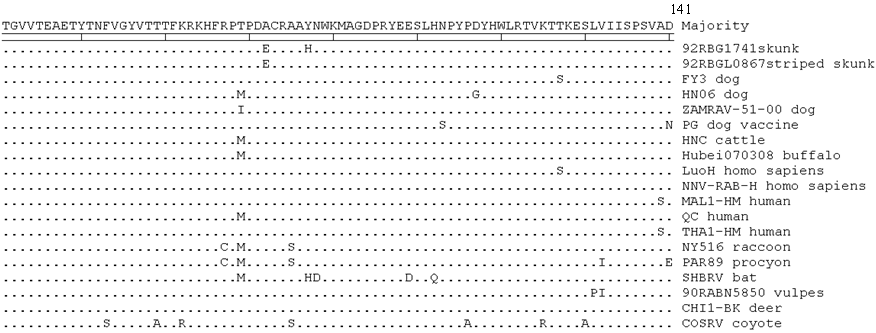


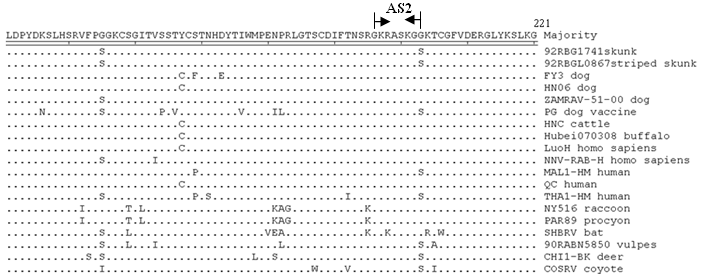


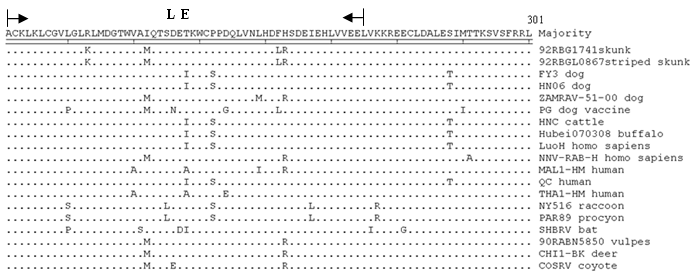


**Fig.3**

**Comparative analysis of G gene amino acid with other RABVs isolated from different animals**. Dots represent identity among all sequences. Arrows mark the range of the signal peptide, antigenic site, linear epitope and endo-domain. Trans-membrane (TM) domain was framed. SP: signal peptides; ENDO: endo-domain; AS2: antigenic site II; AS3: antigenic site III; LE: linear epitope.


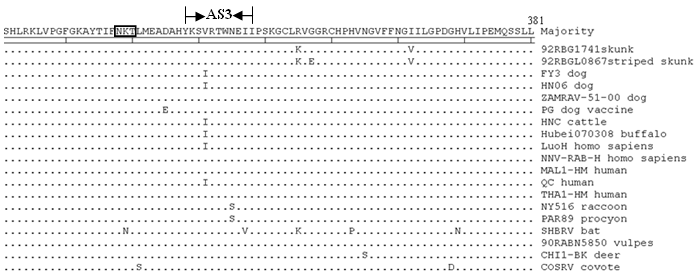


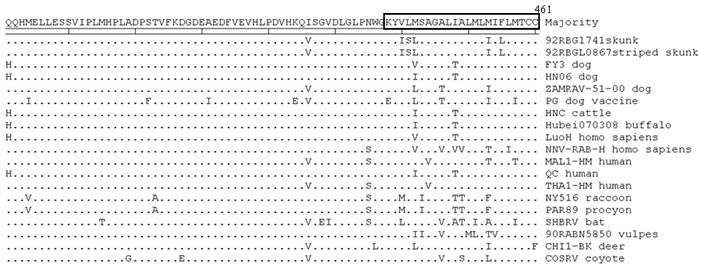


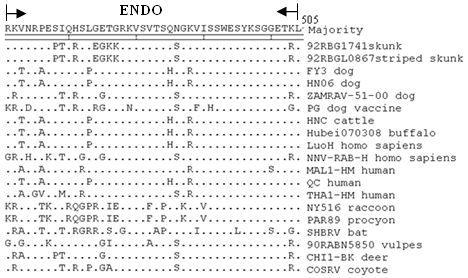


**Fig.3(continued)**

**Comparative analysis of G gene amino acid with other RABVs isolated from different animals**. Dots represent identity among all sequences. Arrows mark the range of the signal peptide, antigenic site, linear epitope and endo-domain. Trans-membrane (TM) domain was framed. SP: signal peptides; ENDO: endo-domain; AS2: antigenic site II; AS3: antigenic site III; LE: linear epitope.
